# Longitudinal changes in glycated haemoglobin following treatment intensification after inadequate response to two oral antidiabetic agents in patients with type 2 diabetes

**DOI:** 10.1111/dom.13694

**Published:** 2019-04-05

**Authors:** Kibum Kim, Sudhir Unni, Diana I. Brixner, Sheila M. Thomas, Cody J. Olsen, Kimberly L. Sterling, Matt Mitchell, Carrie McAdam‐Marx

**Affiliations:** ^1^ Pharmacotherapy Outcomes Research Center and Department of Pharmacotherapy University of Utah Salt Lake City Utah; ^2^ Global Health Economics and Value Assessment, Sanofi Inc. Bridgewater New Jersey; ^3^ Pharmacy Services, Select Health Murray Utah; ^4^ Pharmaceutical Evaluation and Policy Division University of Arkansas for Medical Sciences Little Rock Arkansas

**Keywords:** antidiabetic drug, glycaemic control, observational study

## Abstract

**Aims:**

To identify change in glycated haemoglobin (HbA1c) for 1 year after treatment intensification in patients with HbA1c >53 mmol/mol (7.0%) while on two classes of oral antidiabetic drugs (OADs).

**Material and methods:**

A retrospective cohort study was conducted using a regional health plan claims database for the period January 1, 2010 to March 31, 2017. Patients with type 2 diabetes (T2DM) whose treatment was intensified with insulin, a glucagon‐like peptide‐1 receptor agonist or a third OAD within 365 days of having HbA1c ≥53 mmol/mol (7.0%) on two OADs were included. The HbA1c trajectory for 1 year after intensification was estimated using a mixed‐effects regression model.

**Results:**

The analysis included 1226 patients with a mean ± SD HbA1c at treatment intensification of 74.2 ± 18.7 mmol/mol (8.93 ± 1.7%). HbA1c was higher in the insulin group (74.2 mmol/mol) than in the non‐insulin group (70.6 mmol/mol), as was the HbA1c decrease (*P* < 0.01) over the 1‐year follow‐up, particularly in patients with baseline HbA1c >9%. After intensification, insulin‐ and non‐insulin‐treated patients achieved an average change by month in HbA1c of −4.7 mmol/mol and −2.6 mmol/mol points, respectively. The analysis predicted HbA1c to be the lowest at 6 to 10 months post intensification, depending on intensification treatment and HbA1c at intensification; however, on average, HbA1c remained above 64.0 mmol/mol (8.0%).

**Conclusion:**

In patients with T2DM, intensification following an HbA1c value ≥53 mmol/mol (7.0%) while on two OADs was associated with a significant improvement in glycaemic control. Patients intensified with insulin had a higher baseline HbA1c but greater HbA1c reduction than those intensified with a non‐insulin agent. However, HbA1c remained above 64 mmol/mol (8.0%) overall. Additional opportunity exists to further intensify therapy to improve glycaemic control.

## 1 INTRODUCTION

Type 2 diabetes mellitus (T2DM) affects 8.5% to 9% of the US population.^1^ Of the necessary assessments for comprehensive diabetes management, glycated haemoglobin (HbA1c) has been a primary indicator for the performance of antidiabetic care and a key predictor of long‐term outcomes.^2^ Studies have shown that well‐controlled glycaemic level (HbA1c <53 mmol/mol or 7.0%) reduces the risk of complications.^3^ Conversely, inadequately controlled HbA1c is associated with a significantly higher risk of vascular complications and leads to blindness or premature death.^4^, ^5^ Consequently, patients with poorly controlled diabetes are expected to have worse outcomes. Despite this evidence, ~50% of patients with T2DM fail to attain optimal glycaemic control.^6^

Pharmacotherapy intensification is generally recommended when glycaemic level is inadequately controlled despite acceptable medication adherence.^7^ Supporting the clinical guidelines, observational studies have consistently shown a positive association between the decrease in HbA1c and treatment intensification in patients who did not achieve glycaemic control with metformin (MET)‐based antidiabetic care.^8^, ^9^, ^10^ Time in clinical inertia, that is, the failure to initiate or intensify treatment when indicated, needs to be shortened to increase the likelihood of attaining glycaemic control and lower HbA1c levels.^11^ However, 52% of patients with insufficient response to MET, alone or along with another oral antidiabetic drug (OAD), experienced therapeutic inertia for ≥1 year.^8^

A better understanding of the changes in HbA1c over time following treatment intensification could provide healthcare providers with insights into what to expect in terms of a trajectory and duration of improvement. However, we are unaware of clinical trials that assess outcomes after treatment intensification that are not drug‐specific. Clinical trials also often provide HbA1c data captured during regular follow‐up visits. Administrative claims and laboratory data captured during the course of patient care can help to fill this gap. Evaluation of HbA1c trajectories in administrative data is complex, however, because of factors such as sub‐optimal adherence and inconsistent follow‐up and monitoring. Therefore, observational studies often assess the impact of therapeutic changes on glycaemic control based on a single follow‐up HbA1c reading closest to a target follow‐up date (e.g. 6 or 12 months after treatment change) or based on an average HbA1c over a defined follow‐up period.

There have been notable efforts to address this disadvantage of observational studies. Using a mixed‐effect approach, McAdam‐Marx et al^12^ delineated longitudinal changes in HbA1c following pharmacist‐led medication management intervention. Kazemi et al^13^ assessed the association between HbA1c trajectory and diabetes‐related clinical factors including general types of treatment (e.g. diet, insulin and non‐insulin medications). McCoy et al^14^ assessed HbA1c trajectory over time in patients with controlled glycaemia at baseline. However, the assessment of longitudinal change in HbA1c after intensifying treatment in patients who have failed to maintain glycaemic control while on OAD therapy with two agents has not been assessed. These patients with more progressed disease may have a different response to add‐on therapy than those who are treatment‐naïve or have been on monotherapy.^15^

The first objective of the present real‐world study was to compare glycaemic control as measured by HbA1c before and after the treatment intensification in patients whose HbA1c was ≥53 mmol/mol (7.0%) while on two OADs. The second objective was to project the HbA1c trajectory after the treatment intensification therapy stratified by insulin and non‐insulin medications, which included glucagon‐like peptide‐1 receptor antagonist (GLP‐1RA) agents or a third OAD.

## 2 RESEARCH DESIGN AND METHODS

### 2.1 Data

This was a retrospective observational study of commercially insured patients with T2DM from January 2010 to March 2017. The study was based on medical and pharmacy claims data obtained from SelectHealth, a health plan provider in the intermountain region with ~800 000 enrollees, of whom most are in a commercial plan (84%) and a small proportion in a Medicare plan (3%). The study population was, therefore, relatively young compared to the general T2DM population. The SelectHealth claims data were augmented with HbA1c values obtained through a provider‐incentivized quality improvement programme. Thus, this study has the strength of a claims dataset in terms of access to comprehensive medication use data plus laboratory data to assess clinical outcomes. While other claims datasets are augmented with laboratory data, SelectHealth's quality initiative has resulted in HbA1c data being relatively well documented for this population, reducing the risk of measurement bias attributable to missing data.

### 2.2 Study cohort

The analytical cohort was extracted from the claims for medical and pharmacy services between January 1, 2010 and March 31, 2017. Participants included in this study had received OADs in two different classes, either as two separate drug formulations or as fixed‐dose combinations. The included patients' HbA1c test results had been provided to the health plan by their provider, and all had an HbA1c ≥53 mmol/mol or 7.0% (baseline HbA1c) documented at least 60 days after the first prescription for the second class of OAD but no later than 365 days from the last dispensing of the first class of OAD. Included patients also had a medical claim with a T2DM diagnosis code (International Classification of Diseases [ICD]‐9‐CM, 250.x0 or 250.x2; ICD‐10‐CM, E11x) on at least 2 different days during the 365‐day period before the baseline HbA1c.

**Figure 1:**
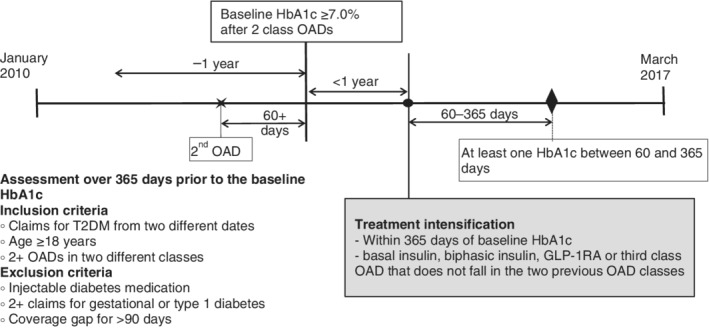
Study timeline. GLP‐1RA, glucagon‐like peptide‐1 receptor agonist; HbA1c, glycated haemoglobin; OAD, oral antidiabetic drug; T2DM, type 2 diabetes

All included patients received treatment intensification for T2DM defined by a pharmacy claim for a basal or biphasic insulin (hereafter referred to as insulin), GLP‐1RA, or a third OAD that was not in one of the two baseline OAD classes. Patients with a >90‐day enrolment gap while on two OADs or before the treatment intensification were excluded from the analytical cohort. Finally, patients needed to have had documented HbA1c values between 60 and 365 days from the treatment intensification date, with 365 days marking the end of the follow‐up period. Patients who had two or more claims for type 1 diabetes or gestational diabetes were excluded, as were patients who received an injectable antidiabetic agent including any insulin, GLP‐1RA or pramlintide during the baseline period and before treatment intensification (Figure 1).

The HbA1c records were captured from 365 days before to 365 days after the date of treatment intensification. The HbA1c measure on the date of intensification or the value closest to that date was considered as the HbA1c at intensification. Clinical characteristics and demographic information including age, gender, type of health plan (i.e. commercial insurance, Medicare and Medicaid), and geographic location by state were captured during the 365 days before treatment intensification. The Diabetes Complication Severity Index (DCSI) was calculated based on diagnoses codes captured during the 365‐day baseline period.^16^

### 2.3 Outcomes

The outcome was the difference in HbA1c before and after the date of treatment intensification.

### 2.4 Covariates

Baseline characteristics were described by treatment intensification group categorized as insulin versus non‐insulin and included age, gender, geographic location by state, type of insurance, HbA1c at baseline and at intensification, baseline OAD treatment, and DCSI. Average proportion of days covered over the baseline period while patients were on two OADs was calculated using Choudhry's prescription‐based method to address adherence to an antidiabetic treatment regimen.^17^

### 2.5 Statistical analyses

Descriptive statistics were used to present baseline characteristics. Statistical significance between groups was tested using Student's *t*‐test or the chi‐squared test depending on the type of variable. Fisher's exact test replaced the chi‐squared test if frequency for any cell was <5.

All HbA1c measures observed over 365 days before and after the treatment intensification were visually examined using a scatter plot to show the density of the HbA1c values over time. The unweighted arithmetic mean of all HbA1c values was compared between the periods using Student's *t‐*test. The trajectory of HbA1c over the 2 years was illustrated using a crude mean for each monthly interval without imputation or deletion.

Post‐intensification HbA1c measures were estimated at monthly intervals based on available HbA1c data using a linear mixed‐effect model, which accounted for non‐standard timing of HbA1c measures after intensification. The model included intercept (i.e. index HbA1c), linear trajectory, curve‐linear trajectory, index HbA1c effect on insulin, and the effect of insulin on both linear and curve‐linear coefficients. The linear and curve‐linear coefficients, respectively, explain the immediate influence of the treatment intensification on the changes in HbA1c and the effect of treatment intensification attenuated over time. Random intercept and random linear functions were included in the model. The analysis was further stratified by HbA1c at intensification of <75 mmol/mol or ≥75 mmol/mol (<9% or ≥9.0%). The HbA1c trajectories for the two subgroups were projected using the fixed effect coefficient estimates. The effect of GLP‐1RAs versus OADs on the HbA1c change was tested in the overall and the subgroup‐specific models.

### 2.6 Institutional review board approval

This study protocol was reviewed and deemed exempt from University of Utah institutional review (#00098483) and was approved by the Intermountain Healthcare institutional review board (#1050483).

## 3 RESULTS

### 3.1 Clinical characteristics at intensification

A total of 1226 patients were included in the analysis (Figure S1). Of these, 54.6% (n = 669) received MET + sulphonylureas (SUs) during the 1 year before insufficient glycaemic control was identified, and 28.3% (n = 347) received MET + dipeptidyl peptidase‐4 (DPP‐4) inhibitors. MET + thiazolidinediones and SUs + DPP‐4 inhibitors accounted for 7.7% (n = 95) and 4.7% (n = 58) of the analytical cohort, respectively. Baseline OAD combinations differed significantly between the insulin and non‐insulin group, with 65.4% in the insulin group receiving MET + SUs versus 51.1% in the non‐insulin intensification group.

The age at treatment intensification was similar in the insulin and the non‐insulin groups, with the mean ± SD age being 54 ± 11 years and 55 ± 10 years, respectively (*P* = 0.19). All patients had HbA1c results provided to the health plan by their provider. The number of HbA1c results reported by providers was essentially equivalent to the number of claims for HbA1c tests, indicating very complete reporting by providers. The number of HbA1c claims and tests did not differ between groups. When assessing HbA1c values, baseline HbA1c was significantly higher in the insulin group, with a mean of 81.1 ± 21.7 mmol/mol (9.55 ± 1.97%) versus 69.3 ± 15.0 mmol/mol (8.48 ± 1.36%) in the non‐insulin group (*P* < 0.01). The median time from the baseline HbA1c date to treatment intensification date was 9 days, and 75% of patients experienced treatment intensification within 35 days from the baseline HbA1c date. HbA1c slightly increased from baseline until treatment intensification, with mean HbA1c at treatment intensification in the insulin and non‐insulin groups of 85.6 ± 15.7 mmol/mol (9.96 ± 1.98%) and 70.7 ± 15.7 mmol/mol (8.61 ± 1.43%), respectively. DCSI differed between groups (*P* < 0.01) with a larger proportion of patients having a DCSI ≥3 in the insulin group (18.1%) than in the non‐insulin group (8.9%). Baseline medication adherence did not differ by treatment intensification, with average proportion of days covered being 0.84 in both groups (Table 1).

**Table 1:** Baseline characteristics by the type of treatment intensification

	Overall N = 1226	Insulin N = 295	OAD or GLP‐1RA N = 931	*P*
Mean (SD) age, years	54.9 (10.3)	54.1 (11.22)	55.1 (10.01)	0.19
Age group, %				
≥65 years	12.9	13.2	12.8	0.98
<65 years	87.1	86.8	87.2	
Men, %	59.2	55.3	60.4	0.37
Geographic region, %				0.78
Utah	95.1	95.3	95.1	
Idaho	3.3	3.4	3.3	
Other	1.5	1.0	1.7	
Type of health plan, %				0.21
Commercial	88.8	86.4	89.6	
Medicare	8.9	10.2	8.5	
Medicaid	2.3	3.4	1.9	
Mean ± SD HbA1c at baseline, mmol/mol (%)	72.1 ± 16.6 (8.74 ± 1.51)	81.1 ± 21.7 (9.55 ± 1.97)	69.3 ± 15.0 (8.48 ± 1.36)	<0.01
HbA1c, %				
≥53 mmol/mol to <75 mmol/mol (≥7 to <9%)	66.5	46.8	72.7	<0.01
≥75 mmol/mol (≥9.0%)	33.5	53.2	27.3	
Mean ± SD HbA1c at intensification, %	74.2 ± 18.4 (8.93 ± 1.67)	85.6 ± 21.8 (9.96 ± 1.98)	70.7 ± 15.7 (8.61 ± 1.43)	<0.01
HbA1c, mmol/mol (%)				
≥53 mmol/mol to <75 mmol/mol (≥7 to <9%)	60.4	33.2	69.0	<0.01
≥75 mmol/mol (≥9.0%)	39.6	66.8	31.0	
Mean ± SD number of HbA1c claims				
On or 1 year before intensification	2.57 (1.11)	2.45 (1.19)	2.61 (1.08)	0.05
1 year after intensification	2.19 (1.17)	2.27 (1.14)	2.17 (1.18)	0.20
Mean ± SD number of HbA1c values reported by provider				
On or 1 year before intensification	2.60 ± 1.10	2.53 ± 1.20	2.62 ± 1.07	0.27
1 year after intensification	2.26 ± 1.16	2.35 ± 1.16	2.24 ± 1.16	0.17
DCSI, %				<0.01
DCSI 0	58.0	50.5	60.4	
DCSI 1	19.3	18.6	19.5	
DCSI 2	11.6	12.9	11.2	
DCSI ≥3	11.1	18.0	8.9	
Two OADs before intensification, %				<0.01
MET + SU	54.5	65.4	51.1	
MET + DPP‐4 inhibitors	28.3	22.7	30.1	
MET + TZDs	7.7	4.1	8.9	
SUs + DPP‐4 inhibitors	4.7	5.1	4.6	
Other	4.7	2.7	5.3	
Mean ± SD PDC on the two OADs	0.84 ± 0.14	0.84 ± 0.15	0.84 ± 0.14	0.67
Conditions in DCSI calculation, %				
Ophthalmic complication (+1)	6.0	9.2	5.0	0.01
Ophthalmic complication (+2)	1.5	1.7	1.5	1.00
Nephropathy (+1)	9.3	13.2	8.1	0.01
Nephropathy (+2)	4.6	7.1	3.8	0.02
Neuropathy	18.7	23.1	17.3	0.03
Cerebrovascular disease (+1)	0.3	0.7	0.2	0.53
Cerebrovascular disease (+2)	1.6	2.4	1.3	0.30
Cardiovascular disease (+1)	10.2%	10.5%	10.1%	0.93
Cardiovascular disease (+2)	6.8%	7.5%	6.6%	0.68
Peripheral vascular disease (+1)	1.7%	2.0%	1.6%	0.82
Peripheral vascular disease (+2)	1.9%	4.1%	1.2%	<0.01
Metabolic disease (+1)	0.2%	0.7%	0.1%	0.29
Metabolic disease (+2)	7.4%	12.5%	5.8%	<0.01
**Year of intensification (column), %**				0.86
2011	11.7	11.5	11.8	
2012	16.3	19.0	15.5	
2013	17.3	17.3	17.3	
2014	17.1	16.6	17.3	
2015	23.3	21.7	23.8	
2016 or 2017	14.2	13.9	14.3	
Year of intensification (row), %				
2011		23.6	76.4	
2012		28.0	72.0	
2013		24.1	75.9	
2014		23.3	76.7	
2015		22.4	77.6	
2016 or 2017		23.6	76.4	

Abbreviations: DCSI, Diabetes Complication Severity Index; GLP‐1RA, glucagon‐like peptide‐1 receptor agonist; MET, metformin; OAD, oral antidiabetic drug; PDC, proportion of days covered; SU, sulphonylurea; TZD, thiazolidinedione.

Note: Data for baseline characteristics were collected during the 1 year before treatment intensification if not otherwise specified.

Complication (+1), the number of patients having a record of the condition over the 1 year prior to the date of intensification that adds one point to the DSCI calculation; Complication (+2), the number of patients having a record of a severe complication that adds two points to the DSCI calculation.

### 3.2 HbA1c trajectory: descriptive analysis

**Figure 2:**
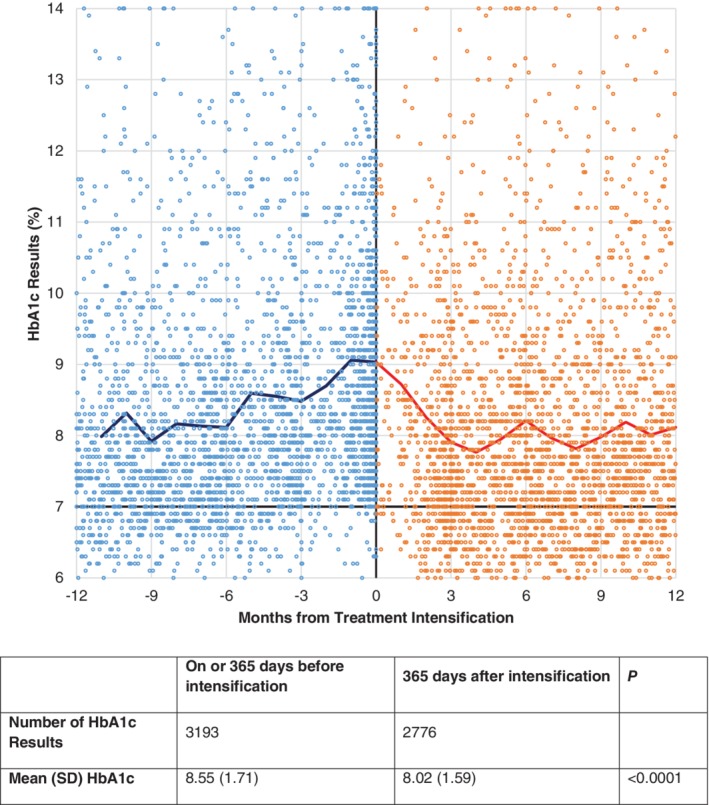
Changes in glycated haemoglobin (HbA1c) before and after the treatment intensification, scatter plot and mean of available HbA1c by month

The number of HbA1c values included in this analysis was 5969, of which 3193 were on or before treatment intensification and 2776 were after the treatment intensification. The mean HbA1c in the overall cohort was 7.01 ± 18.8 mmol/mol (8.55 ± 1.71%) before the treatment intensification and 64.2 ± 17.5 mmol/mol (8.02 ± 1.59%) after the treatment intensification (*P* < 0.0001; Figure 2). When averaged by month, HbA1c mean fluctuated but continuously increased until the treatment intensification date and then decreased from 75.3 mmol/mol (9.03%) at intensification to 61.4 mmol/mol (7.76%) at 4 months after intensification. The monthly average HbA1c thereafter fluctuated between 61.4 and 66.1 mmol/mol (7.76 and 8.19%).

### 3.3 HbA1c trajectory: mixed‐effect regression model

The results of the regression analyses estimated that HbA1c at intensification was 14.3 mmol/mol (1.30%) higher in the insulin arm compared to the non‐insulin arm. Insulin was also associated with a greater reduction in HbA1c (−2.2 mmol/mol or −0.20%) relative to non‐insulin. This represents the average estimated change per month of −4.8 and −2.4 mmol/mol (−0.44 and −0.24%) for the insulin and non‐insulin groups, respectively. While the estimated HbA1c continued to decline over the follow‐up period in the insulin group until months 6–10, the rate of HbA1c change significantly diminished over time.

**Figure 3:**
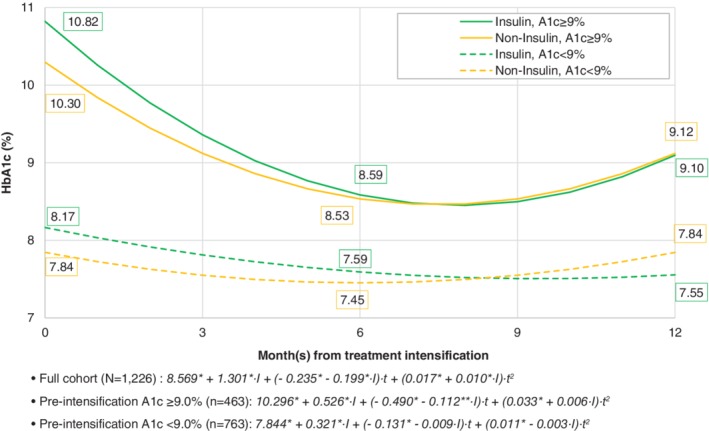
Changes in glycated haemoglobin (HbA1c) by treatment intensification (insulin vs. oral antidiabetic drugs [OAD] or glucagon‐like peptide‐1 receptor agonists [GLP‐1RAs]), HbA1c trajectories from subgroup analysis for insufficient (<75 mmol/mol or 9.0%) and poorly (≥75 mmol/mol or 9.0%) controlled HbA1c before treatment intensification. Note. *t*, temporal distance in month(s) from the date of treatment intensification; *I* = 1 for patients receiving insulin for the intensification; *I* = 0 for patients receiving GLP‐1RAs or OADs for the intensification; ^*^ *P* < 0.05, ^**^ *P* < 0

Figure 3 shows the estimated HbA1c change over the 1‐year post‐intensification period by treatment group and HbA1c at intensification. The covariance between HbA1c at treatment intensification and monthly change in HbA1c was −5.4 mmol/mol (−0.49%) in patients with HbA1c <75 mmol/mol (9.0%) at intensification and −5.5 mmol/mol (−0.50%) in those with HbA1c ≥75 mmol/mol (9.0%). These data indicate that a greater HbA1c reduction was observed in patients with higher HbA1c at intensification, overall. Insulin was associated with a higher HbA1c at intensification and a greater reduction over the follow‐up period than intensification with a non‐insulin agent. For all patients with HbA1c ≥75 mmol/mol (9.0%) at intensification and non‐insulin patients with HbA1c <75 mmol/mol (9.0%) at intensification, HbA1c reduction levelled off between months 6 and 8, then began to rise. Insulin patients with HbA1c <75 mmol/mol (9.0%) at intensification experienced a modest drop in HbA1c until month 9, then HbA1c stabilized. No significant difference in HbA1c change was identified between patients whose therapy was intensified with GLP‐1RAs versus OADs (Table S1 and Figure 2).

## 4 DISCUSSION

The primary goal of diabetes management is to maintain a level of blood glucose that balances the risk of complications with the risks associated with treatment, including hypoglycaemia.^7^ With disease progression, glycaemic control is maintained by a stepwise addition of antidiabetic agents, after patients fail to achieve HbA1c with initial treatment, often MET monotherapy.^7^ Previous research by Fu and Sheehan^8^ has shown that 50% of patients who did not sufficiently respond to MET treatment as monotherapy or in combination with other oral agents required and received treatment intensification within 1 year. The authors also identified that the likelihood of better HbA1c maintenance increased when treatment inertia was shorter than 6 months.^8^, ^9^

The present study expands on the research by Fu and Sheehan^8^, ^9^ by not limiting prior therapy to MET users, and by specifically focusing on patients who had failed to maintain glycaemic control while on two OADs. We found that switching or adding a third agent significantly reduced HbA1c. The decrease in the HbA1c was greatest over the first 4 months, and HbA1c for the overall cohort remained below HbA1c at intensification for the follow‐up period. HbA1c started to increase before the end of the first year after the intensification. This could be for multiple reasons, including disease progression or reduced adherence, and warrants further investigation.

Overall, glycaemic response was greatest in those with poor glycaemic control, which was similarly observed in previous observational studies. In a study by Pantalone et al including patients whose HbA1c remained >53 mmol/mol (7.0%) on MET monotherapy, the level of improvement in glycaemic control was linearly associated with an increase in the baseline HbA1c level.^11^ In another recent analysis of insurance claims for patients with T2DM, where treatment failure was defined by ≥64 mmol/mol (8.0%) after a treatment with metformin monotherapy or in combination with other OADs, the change in HbA1c was greater with a higher pre‐intensification HbA1c. From the same study, treatment intensification in patients having pre‐index HbA1c ≥75 mmol/mol (9.0%) resulted in the HbA1c change of −23.7 mmol/mol (−2.15%), which is close to our estimate of HbA1c decrease in the poor glycaemic control group.^9^

The HbA1c reduction was also 2.2 mmol/mol (0.2%) greater per month for those prescribed insulin after failing two OADs relative to those prescribed a non‐insulin agent; however, patients in the insulin group also had higher HbA1c levels at the time of intensification. This observation is consistent with American Association of Clinical Endocrinologists and American Diabetes Association guidelines in place at the time of the study, which encourage the use of insulin when HbA1c exceeds 75 or 86 mmol/mol (9.0% or 10%).^7^, ^15^, ^18^ It also reflects the demonstrated glycaemic response to insulin.^7^

The present study is important in providing data on the effectiveness of diabetes therapy intensification after failing to maintain glycaemic control on two OADs. The American Association of Clinical Endocrinologists and American College of Endocrinology consensus statement has highlighted that patients taking two or more medications will probably see less improvement with intensification or a switch to a new agent than can be expected when the same agent is used as first‐ or second‐line therapy.^15^ Observational studies have also seen a negative association between the number of prior classes used and the level of HbA1c reduction.^12^ This observation may reflect the decline in insulin sensitivity and β‐cell function with T2DM progression.^19^ Despite the possibility of attenuated efficacy, the average patient in the present study prescribed a third line of therapy after two OADs experienced a clinically important improvement in glycaemic control.

The analytical approach used in the present study also contributes to the literature in terms of its design. In the real‐world setting, patients are not monitored on a set schedule as in a clinical trial; therefore, identifying HbA1c patterns at defined intervals from observational data is challenging as missing data necessitates the use of imputation or leads to attrition of patients from data analysis. When all HbA1c data are used, different periods of time from initiation of a new agent to measurement may lead to bias. As such, observational studies usually assess outcomes based on a single follow‐up HbA1c at a defined point in time, a mean HbA1c over the follow‐up period or the attainment of glycaemic goal at any time during the follow‐up period.^20^, ^21^, ^22^ Thus, they provide an estimate of glycaemic control after a specific period post an index treatment. We used a mixed‐effect regression model, which addresses potential bias, including correlation between repeated measures, and accounts for the non‐linear nature of HbA1c over time.^23^ Using this model, we were able to simulate the average HbA1c over a 1‐year time period, providing a more realistic view of maximum efficacy and 1‐year treatment durability in a real‐world setting.

Clinical practice should be informed by the present findings. While insulin therapy was associated with the greatest reduction in HbA1c for all patients with HbA1c ≥75 mmol/mol (9.0%), those with HbA1c ≥75 mmol/mol (9%) and receiving intensification with a GLP‐1RA or third OAD also experienced significant reductions in HbA1c over the first 6 months. Despite these gains, patients on average did not attain HbA1c <64 mmol/mol (8.0%). This is a conservative goal for the study population, given their younger age and that >50% of the population did not have evidence of diabetes‐related complications according to the DCSI. There is therefore room for improvement. Obviously, failure to attain goal should also serve as a trigger for diabetes education and medication management efforts to optimize drug therapy and address issues with patient self‐management, including adherence to medications and lifestyle recommendations.

This study was subject to the inherent limitations faced by studies using administrative claims databases. Clinical data beyond HbA1c, lifestyle behaviours, and more detailed demographic information that could influence treatment selection and outcome were not available. Future work may integrate claims and electronic medical records to provide comprehensive clinical information. An integrated dataset with matching or other methods to balance comparison groups and control for more potential confounders might be considered.

The HbA1c difference observed between the insulin and non‐insulin groups may have introduced bias by confounding. While the stratified analysis based on HbA1c at intensification partially addressed this limitation, it did not control for the effect of disease severity on follow‐up HbA1c trajectories. In addition, this study captured treatment intensification based on a prescription claim for a different class of diabetes drug, but did not distinguish whether this treatment represented an add‐on or switch, nor did it assess for dose escalations after intensification. Finally, the study did not control for diabetes medication compliance in the regression analyses. Baseline adherence did not differ, however, between intensification groups. The role of post‐intensification compliance on HbA1c trajectories should be addressed in future studies.

In conclusion, treatment intensification after failing to achieve glycaemic control on two OADs was associated with a significant reduction in HbA1c within 6 months that was maintained or just slightly rose after 6 to 9 months. The largest reduction in HbA1c was seen in those with HbA1c ≥75 mmol/mol (9.0%) and treated with insulin, but a significant improvement in glycaemic control was also seen with non‐insulin agents. Even with improvement in glycaemic control, many patients failed to achieve an HbA1c target <64 mmol/mol (8.0%). Thus, opportunity exists for additional and/or more aggressive treatment intensification and patient engagement and management to achieve optimal glycaemic control and help reduce the risk of diabetes complications.

## CONFLICT OF INTEREST

K.K., S.U., C.O., C.M.‐M. and M.M. have no conflicts of interest to declare. S.M.T. is an employee and a stock/shareholder of Sanofi, Inc. K.L.S. was an employee and a stockholder of Sanofi, Inc. at the time this work was conducted. D.B. has served as an advisory board member and presenter for Sanofi, Inc.

## AUTHOR CONTRIBUTIONS

The study concept was proposed in collaboration by all authors. K.K. and C.M.‐M. developed the research design and analysis plan. The research design and analysis plan were reviewed and modified by all coauthors. C.O. and M.M. contributed to the data acquisition. K.K. conducted the cohort extraction and statistical analysis. K.K. and S.U. compiled the draft manuscript. All co‐authors materially participated in this manuscript preparation. The overall research project was supervised by D.B. and C.M.‐M.

## PRIOR PEER‐REVIEWED PRESENTATION AT A PROFESSIONAL/SCIENTIFIC CONFERENCE

Part of the results of this study was presented at the American Diabetes Association meeting in Orlando, Florida, June 22–26, 2018.

## Supporting information


**APPENDIX Table 1** Baseline characteristics, GLP‐1RA vs. OAD
**APPENDIX Figure 1.** Cohort Selection Flow
**APPENDIX Figure 2.** Changes in A1c by Treatment Intensification – Insulin, OAD and GLP‐1RA.
