# Supplementary material for: Longitudinal changes in glycated haemoglobin following treatment intensification after inadequate response to two oral antidiabetic agents in patients with type 2 diabetes
Source: Diabetes Obes Metab. 2019 Apr 5;21(7):1725–33. doi: 10.1111/dom.13694 (PMC6618330; doi:10.1111/dom.13694)
Supplement: Supplementary file 1 — APPENDIX Table 1 Baseline characteristics, GLP‐1RA vs. OAD APPENDIX Figure 1. Cohort Selection Flow APPENDIX Figure 2. Changes in A1c by Treatment Intensification – Insulin, OAD and GLP‐1RA. [file DOM-21-1725-s001.docx]

**APPENDIX Table 1. Baseline characteristics, GLP-1RA vs. OAD**

| **Mean (SD) / %** | **GLP-1RA N=199** | **OAD N=731** | **p-value** |
| --- | --- | --- | --- |
| **AGE (mean, SD)** | 52.24 (10.34) | 55.47 (9.98) | <0.01 |
| ≥65 | 92.0% | 85.7% | 0.03 |
| <65 | 8.0% | 14.3% |  |
| **Male - Gender** | 51.8% | 62.7% | 0.04 |
| **Geographic region** |  |  | 0.15 |
| Utah | 95.0% | 95.1% |  |
| Idaho | 2.0% | 3.7% |  |
| Other | 3.0% | 1.4% |  |
| **Type of Health Plan** |  |  | 0.04 |
| Commercial | 94.5% | 88.3% |  |
| Medicare | 4.5% | 9.6% |  |
| Medicaid | 1.0% | 2.2% |  |
| **A1c at Baseline (%)** |  |  |  |
| Mean (SD) | 8.42 (1.27) | 8.49 (1.39) | 0.48 |
| A1c ≥7- <9% | 72.9% | 72.7% | 1.00 |
| A1c ≥9.0% | 27.1% | 27.3% |  |
| **A1c at Intensification (%)** |  |  |  |
| Mean (SD) | 8.68 (1.32) | 8.58 (1.45) | 0.37 |
| A1c ≥7- <9% | 64.3% | 70.3% | 0.05 |
| A1c ≥9.0% | 35.7% | 29.8% |  |
| **DCSI*** | 0.60 (1.04) | 0.59 (1.02) | 0.89 |
| DCSI 0 | 64.3% | 65.3% | 0.98 |
| DCSI 1 | 21.1% | 19.8% |  |
| DCSI 2 | 9.0% | 9.0% |  |
| DCSI ≥ 3 | 5.5% | 5.9% |  |
| **2OADs before Intensification** |  |  | 0.317 |
| BG+SU (%) | 50.1% | 54.8% |  |
| BG+DPP4I (%) | 31.1% | 26.1% |  |
| BG+TZD (%) | 9.0% | 8.5% |  |
| SU+DPP4I (%) | 4.1% | 6.5% |  |
| Other (%) | 5.6% | 4.0% |  |
| Average PDC on the 2OADs | 0.83 (0.15) | 0.84 (0.14) | 0.30 |
| **Conditions in DCSI^†^ calculation** |  |  |  |
| Ophthalmic complication (+1) | 5.0% | 4.8% | 1.00 |
| Ophthalmic complication (+2) | 0.5% | 1.2% | 0.57 |
| Nephropathy (+1) | 8.5% | 9.8% | 0.68 |
| Nephropathy (+2) | 5.5% | 4.5% | 0.35 |
| Neuropathy | 18.6% | 16.0% | 0.44 |
| Cerebrovascular Disease (+1) | 1.5% | 1.2% | 0.73 |
| Cerebrovascular Disease (+2) | 1.5% | 1.1% | 0.77 |
| Cardiovascular Disease (+1) | 8.5% | 11.5% | 0.30 |
| Cardiovascular Disease (+2) | 3.5% | 4.6% | 0.50 |
| Peripheral Vascular Disease (+1) | 3.0% | 2.3% | 0.61 |
| Peripheral Vascular Disease (+2) | 3.0% | 1.4% | 0.13 |
| Metabolic Disease (+1) | 0.5% | 0.5% | 1.00 |
| Metabolic Disease (+2) | 0.5% | 0.3% | 0.70 |
| **Year of Intensification (Column%)** |  |  | 0.10 |
| 2011 | 8.5% | 12.7% |  |
| 2012 | 17.6% | 14.9% |  |
| 2013 | 21.6% | 16.1% |  |
| 2014 | 14.6% | 18.0% |  |
| 2015 | 20.6% | 24.7% |  |
| 2016 or 2017 | 17.1% | 13.5% |  |
| **Year of Intensification (Row %)** |  |  |  |
| 2011 | 15.5% | 84.5% |  |
| 2012 | 24.3% | 75.7% |  |
| 2013 | 26.7% | 73.3% |  |
| 2014 | 18.0% | 82.0% |  |
| 2015 | 18.5% | 81.5% |  |
| 2016 or 2017 | 25.6% | 74.4% |  |

Note. Data collected over one year before the treatment intensification if not specified.

**^†^** DCSI, Diabetes Complication Severity Index

Complication (+1), the number of patients having a record of the condition over the 1 year prior to the date of intensification that adds one point to the DSCI calculation; Complication (+2), the number of patients having a record of a severe complication that needs to add two points to the DSCI calculation.

Abbreviation/Acronym: Insulin, treatment intensification with basal or biphasic insulin; GLP-1RA, treatment intensification with GLP-1RA without basal or biphasic insulin; OAD, treatment intensification with a 3^rd^ class OAD without injectable antidiabetic agent; PDC, Proportion of Days Covered

**APPENDIX Figure 1. Cohort Selection Flow**

N = 51,239

N = 24,945

N=3,739

N = 15,127

T2DM patients with any HbA1c ≥7.0

potentially eligible to be a baseline A1c

Exclude if a patient had more than one record of GDM or T1DM over 365days prior to the potential baseline A1c

Continuous Enrollment^†^,

1 year before the potential baseline HbA1c date

OADs in two classes for 365 days prior to the date of HbA1c ≥7.0%

Patients with treatment intensification including

basal/biphasic insulin, GLP-1RA and 3^rd^-class OAD

within 365 days from the baseline A1c date

N=1,403

Analytic Cohort

N= 1,226

Patients with treatment intensification and outcome HbA1c value

on 60 – 365 days from the intensification,

Continuous enrollment^†^, 1 year before the intensification (0 exclusion)

Insurance Claims between 01/01/2010 – 03/31/2017

Commercial: 1,476 K; Medicaid: 224K; Medicare: 52K

^†^Continuous enrollment, There was no or less than 90-day coverage gap.

**APPENDIX Figure 2. Changes in A1c by Treatment Intensification – Insulin, OAD and GLP-1RA**

|  |
| --- |
| - Model : *8.562*^*^ *+ 1.308*^*^*·I + 0.033*^*^*·G + (- 0.224*^*^ *- 0.210*^*^*·I + -0.054*^*^*·G)·t + (0.016*^*^ *+ 0.011*^*^*·I + 0.006**^*^*·G)·t^2^* |

Note. *t*, temporal distance in month(s) from the date of treatment intensification; *I*=1 for patients receiving insulin for the intensification; *G*=1 for patients receiving GLP-1RA for the intensification, *I*=0 and *G*=0 for patients receiving OAD for the intensification;  ^*^ p<0.05, ^**^ p<0.1
